# Exertion Perception When Performing Cutting Tasks in Poultry Slaughterhouses: Risk Assessment of Developing Musculoskeletal Disorders

**DOI:** 10.3390/ijerph17249534

**Published:** 2020-12-19

**Authors:** Adriana Seára Tirloni, Diogo Cunha dos Reis, Salvador Francisco Tirloni, Antônio Renato Pereira Moro

**Affiliations:** 1 School of Technology, Federal University of Santa Catarina, Florianópolis, Santa Catarina 88040-370, Brazil; salvador@tirloni.com.br (S.F.T.); renato.moro@ufsc.br (A.R.P.M.); 2 Biomechanics Laboratory, Center of Sports (CDS), Federal University of Santa Catarina, Florianópolis, Santa Catarina 88040-370, Brazil; diogo.biomecanica@gmail.com

**Keywords:** slaughterhouse, meat-packing industry, risk assessment, ergonomics, muscular disorders, OCRA method

## Abstract

Brazil is the leader in poultry meat exports, in which most products are in the form of cuts. This study analyzed the exertion perception of poultry slaughterhouses workers when performing cutting tasks, as well as the influence of knife sharpness on the risk of developing musculoskeletal disorders by Occupational Repetitive Action (OCRA) method. Participants (*n* = 101) from three slaughterhouses were asked to rate their perceived exertion on the Borg scale during the cutting task when the knife was well and poorly sharpened. The OCRA results showed that the score for cutting with a dull knife was greater (43.57 ± 13.51) than with a sharp knife (23.79 ± 3.10) (*p* < 0.001). Consequently, there was a significant increase in the risk level of acquiring upper-limb work-related musculoskeletal disorders (UL-WMSD) by using a “poorly sharpened” knife (29%; *p* < 0.001; Borg scale 2–8). Thus, maintaining well-sharpened knives for optimal performance of the cutting task (fewer technical actions) is suggested, as well as including knife sharpening in the standard operating procedure to reduce musculoskeletal disorders.

## 1. Introduction

The Brazilian Association of Animal Protein stated that Brazil was the leader in poultry meat exports and the third largest producer in the world in 2019 [1]. In contrast, from 2017 to 2018, this economic activity changed from third to second place in accidents due to occupational diseases, and overall, synovitis and tenosynovitis was the second highest incidence cited by the International Classification of Diseases (ICD-11) in this country [2].

According to an annual report, the type of product that led the exports were the cuts (67%) [1], that is, tasks were performed by machinery and/or workers using a hand tool. In poultry processing jobs, there are several risk factors present to develop upper-limb work-related musculoskeletal disorders (UL-WMSDs): repetition, force, awkward and static postures, vibration and cold [3]. The use of a knife and gloves [4,5] along with the increasing levels of hand activity and force [6,7] were associated with WMSDs. One of the factors that cause UL-WMSDs is high force exertions or mechanical compression of tissues, especially in the hands [8]. Poultry slaughterhouse workers perform heavy exertions, whether to carry out a task or to maintain control of equipment or tools [3].

Regardless, workers must also wear gloves to protect against cuts and/or cold, which may exacerbate musculoskeletal constraints [4], increase muscle activity, wrist deviation, and discomfort whilst reducing hand grip strength, forearm torque strength and touch sensitivity [9]. Furthermore, slaughterhouse workers are subjected to cold environments. As stated by Ramos et al. [10] and Tirloni et al. [11], most slaughterhouse workers presented at least one finger with a temperature below 24 °C and felt cold in their hands, despite wearing gloves. In addition, the chance of feeling cold in the hands for a worker who used a tool was greater than for a worker who did not [11]. Likewise, slaughterhouse workers that perform tasks in cold environments and feel cold are more likely to experience bodily discomfort [12].

There is the challenge of assessing the force exerted during cutting tasks. However, cutting force analyses were performed using instrumented knives [13,14,15,16]. Nevertheless, these knives had a wire and the investigations were conducted in a laboratory [16,17,18,19] in slaughterhouses [14,15,20,21,22] or in both environments [13,23]. There were factors that could potentially interfere with the results of these studies, as data collection was executed in an unrealistic work context.

Conversely, to overcome this difficulty of assessing the force exerted by the worker without specific tools (direct measure), the OCRA method [5] employs the Borg CR-10 subjective scale [24]. Besides, researchers have proposed a protocol that could improve the strength of correlations between direct measures of grip force and perceived exertion ratings [25], but investigations about the cutting force of slaughterhouse workers using this scale are scarce [26]. Blade sharpness causes positive effects in force exposure of the workers [20,27], however, analyses disagree with these outcomes [15,21].

Although the need and importance of knife sharpening used by meat cutters to prevent WMSD had been proven [28,29,30], as well as the recommendation to apply the OCRA method to assess the handling of low loads at high repetition frequency in slaughterhouses [31], no findings were attained that evaluated the influence of knife sharpness and the development of UL-WMSD by this method. Furthermore, no research examined the exertion perception of slaughterhouse workers regarding the force employed while cutting with a sharp or dull knife.

Therefore, the objective of this research was to analyze the exertion perception of poultry slaughterhouse workers when performing cutting tasks, as well as the influence of knife sharpness on the risk of developing musculoskeletal disorders.

## 2. Method

This cross-sectional study received ethical approval from the Federal University of Santa Catarina, protocol n° 2098/2011. Data were collected in 2019 from three poultry slaughterhouses in the south of Brazil, with a daily working time of 8 h 48 min, excluding 60 min for a meal break, which included: two work shifts; workers performing 453 min of repetitive work per workday; 3 × 20 min of rest breaks and 15 min for uniform change. Other work organizational characteristics are described in Table 1.

The participants worked in artificially cold (≈12 °C) and natural environments. Workers used personal protective equipment for the hands (nitrile, chainmail, cut protection, thermal-protection and polyethylene gloves), besides clothing, aprons, socks and boots provided by the slaughterhouses with a Certificate of Approval from the Brazilian Ministry of Labor.

### 2.1. Participants

The selection method of the slaughterhouses was intentional, but the workers were designated randomly. In the cutting room (cold) or evisceration sector (natural), the researcher selected the first worker at the table or line, skipped one and called the next and so on. As for the eligibility criteria, workers must have used a knife to perform their tasks, already completed the three months training period, and consented to being interviewed and filmed. All invited workers agreed to participate in the study.

Current research included 101 poultry slaughterhouse workers from 18 cutting tasks, 76 women (18–55 years) and 25 men (19–59 years) that had been employed in the company from 3 months to 22 years (2.5 ± 3.1 years).

### 2.2. Instruments

In the workplace, workers were interviewed about individual factors (age and length of time working at the company), work organization (job rotation and work pace on the day of collection), glove use (number), perception of bodily discomfort of the upper limbs and exertion when cutting the chicken, as well as feeling and perceiving cold in their hands. As in the present study, the OCRA method was used to verify the risk of UL-WMSDs, which considers the subjective perception of effort. Besides, the Borg scale 0–10 presented in Colombini and Occhipinti [5] was adapted and applied by the authors (Table 2).

**Figure 1:**
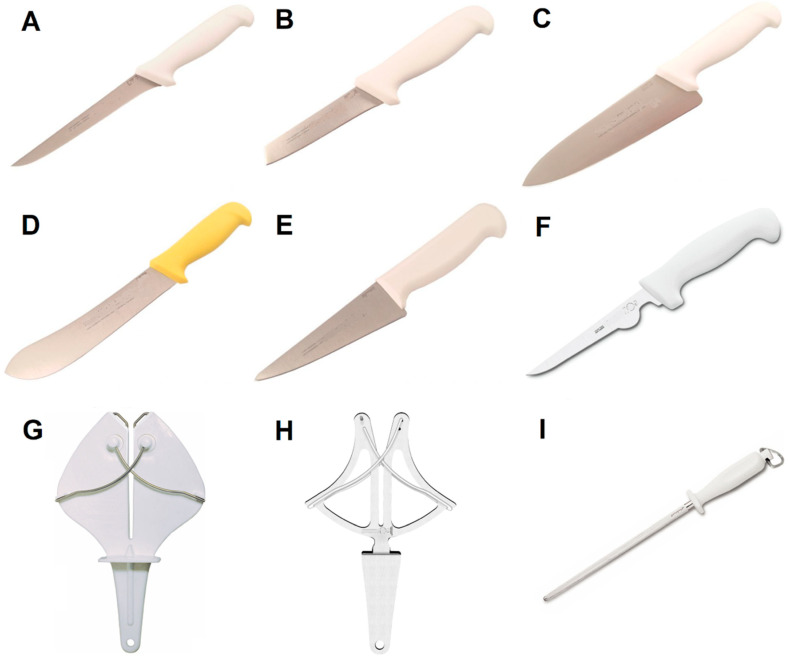
Knives used by workers: (**A**) slaughterhouse 1, cleaning breast and deboning leg; slaughterhouse 2, cleaning breast and cleaning breast automatic; (**B**) slaughterhouse 1, deboning leg; (**C**) slaughterhouse 2, removing neck; (**D**) slaughterhouse 2, removing back and cutting kakugiri; slaughterhouse 3, chicken slaughter; (**E**) slaughterhouse 2, deboning whole leg, removing wing and deboning sassami; slaughterhouse 3, removing wing, removing breast/removing condemnations, cleaning breast, cleaning leg and removing back; (**F**) slaughterhouse 3, deboning back) and knife sharpening accessories (**G**) slaughterhouse 1; (**H**) slaughterhouse 2 and 3; (**I**) slaughterhouse 2 and 3.

According to the health and safety team of the slaughterhouses, each worker received clean and sharp knives (by a professional sharpener) four times per work shift, including knife sharpening accessories at the workstations to sharpen when necessary (Figure 1). To record the frequency of technical actions and bodily postures adopted during the daily work, a camcorder Sony HDR-XR160 (Sony, Tokyo, Japan) was used. As each slaughterhouse has its specific characteristics, product mix, tools used, etc., it was found that six types of knives were utilized by workers in slaughterhouses. The type of knife, the tasks performed with the tool and the respective slaughterhouse are detailed in Figure 1.

The workers were asked about bodily discomfort in the last 12 months, in which the following symptoms were considered: pain, fatigue, shocks, cracks, numbness, tingling, weight, strength loss and movement limitation in upper limbs [32]. Additionally, the workers were questioned about whether they perceived their hands as cold, as well as felt cold in the hands.

### 2.3. OCRA Checklist Method

**Figure 2:**
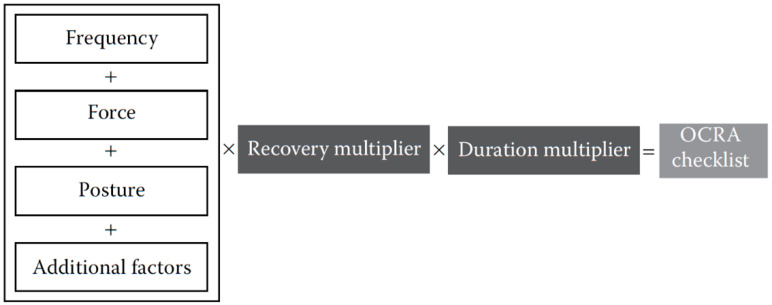
Calculation of risk factors of the OCRA Checklist method.

The method confirms workers’ exposure to risk factors for developing UL-WMSDs as repetitive task duration (duration multiplier), lack of recovery periods (recovery multiplier), action frequency, force demand, inappropriate postures/stereotyped movement (identical actions repeated), and other additional risk factors [5] (Figure 2).

#### 2.3.1. Action Frequency Factor

Technical actions are not the individual movements of the hand, wrist, elbow, or shoulder but rather the overall movement accomplished by one or more joint segments enabling a simple work element to be performed, such as grasping, positioning and so on [5]. Workers were filmed for a minimum of one minute, an average of 10 cycles of each task. Technical actions were sporadically carried out by a worker to sharpen the knife (within the cycle with knife sharpener) at the workstation. Because of this, these actions were not considered to define the frequency factor. The worker sharpened the knife when he/she thought it was necessary. So, there was no guidance from the managers telling them when to sharpen the knife after cutting a certain number chicken pieces.

The number of technical actions in each cycle was established, then the action frequency was calculated (actions/min). The higher the frequency of technical actions per minute, the higher the score of this risk factor (0–10). Work cycles of less than 30 s are considered high repeatability tasks [33].

#### 2.3.2. Force Factor

The OCRA method recommends that the evaluators identify the technical actions in the cycle that involve force, after questioning the worker about the perception of the strength level for each of these technical actions using the Borg scale (0–10) [5]. In addition, the analyst then indicates the duration of each action as a fraction of the duration of the whole cycle. Table 3 presents the determination of the force factor score based on the Borg scale and on the fraction of the time relationship. The duration of the cutting actions (%) throughout the cycle was verified (by videos) to determine the score of the force factor.

#### 2.3.3. Awkward Posture Factor

Upper limb postures are described and assessed based on a representative cycle of each task. The joints of the shoulders, elbows, wrists, hands and fingers were evaluated individually, each joint received a score that could vary between 0–24 depending on the exposure time or doubling the value if the hands were above the head. However, of all the scores calculated for the different joints, only the highest was chosen, and added to the score for stereotype, if applicable, in which the total would be the score for the posture factor.

Conforming to Colombini and Occhipinti [5], when the grip is not optimal for knife use, an intermediate score of 1 may be added (≈one-third of the time), 2 (≈two-thirds of the time) and 3 (≈the entire time). Besides using these scores in the present study, it was also assigned a score of 4 when the worker temporarily held the knife and manipulated the product simultaneously (almost the total cycle time).

In relation to stereotypy, in cycles with a time between 8 s and 15 s or when two-thirds of the entire task is made up of identical technical actions: the score is 1.5. Therefore, with a cycle time less than 8 s or when practically the entire task is composed of identical technical actions: the score is 3 [5].

#### 2.3.4. Additional Risk Factors

Physical-mechanical and organizational risks form two blocks of additional factors that increase the risk. Each risk block should be analyzed separately and for each side of the body. If the risk factor is present in the task, a score of 1 to 4 is attributed, but only one answer is given in each of the two blocks. Physical factors: shocks and counter-shocks for over half the time (score 2), repeated impacts by the hand (the hand is used as a tool) (score 2), use of vibrating tools for almost 1/3 of the time (4) or when there are more additional factors for almost the entire time (3). Additional organizational factors: when there are “buffers” by which the working pace may be slower (1) or the work pace is completely determined by the machine (2). The sum of the partial scores entered in the two blocks generates the final score of an additional factor [5].

#### 2.3.5. Recovery Multiplier

With the purpose of calculating the lack of recovery time risk factor, one should check the number of actual breaks (recovery periods) during the work shift, with a duration of at least 8 min (excluding meal break). It should be noted that there must be a ratio of 5:1 between work time (repetitive tasks using the upper limbs) and recovery time (upper limbs inactive) [5]. When it occurs, the work time is considered to have been recovered (does not receive a score).

**Figure 3:**
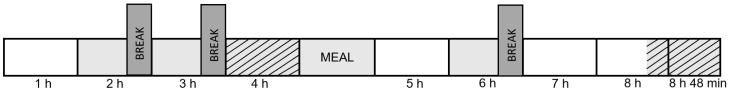
Distribution of the psychophysiological rest breaks in the 8 h 48 min shift.

This method assigns a score of 1 for each hour without adequate recovered time (8–10 min) and 0.5 for 20–40 min working without pause. In addition, the hour before the meal break and the last hour of the work shift are considered automatically recovered hours (shaded rectangle) (Figure 3).

The workers performed 3 psychophysiological breaks of 20 min, well distributed in a work shift of 8 h 48 min. After analyzing the quantity, duration and distribution of breaks during the workday, and discounting 15 min for uniform change, it was identified that 3.5 h were not recovered (white rectangles—1st, 5th, 7th and ½ of the 8th hour) (Figure 3). According to the table provided by the OCRA method [5], for this quantity of hours not recovered (3.5 h), the recovery multiplier score is 1.265.

#### 2.3.6. Duration Multiplier

The organization of the work must be checked to identify the net time of the repetitive task and determine the multiplier factor of the OCRA Checklist deducting the pause times and/or non-repetitive work from the duration of the work shift [5]. The repetitive work time for all slaughterhouses was 453 min and the duration multiplier was 1 (421–480 min).

### 2.4. Knife Sharpness Evaluation

For the knife sharpness evaluation, the recall method was applied in which workers were interviewed about the perceived exertion when cutting the poultry meat in two conditions: with a sharp knife and a dull one. After viewing the Borg scale (Table 2), each participant was instructed to remember and indicate their perceived exertion when he/she was using a well and poorly sharpened knife. It was explained to the worker that the cutting exertion in an extremely hard condition (10 points) was the one in which the worker applied his/her force to cut at almost maximum capacity.

The state of a “well-sharpened” knife was the condition that, in the worker’s perception, the tool required a minimum exertion to perform the cut, and “poorly sharpened” was when the knife was dull, not sharp, and no longer provided the ability to cut easily. In this study, the modus operandi of production and knife sharpening were not changed in the slaughterhouses.

### 2.5. Statistics

The statistical analysis was performed using IBM SPSS Statistics, version 21.0 (IBM Corp., Armonk, NY, USA). Data distribution was tested, so the Wilcoxon test was employed to compare the values of the OCRA Checklist in the two knife sharpness conditions, as well as the scores of the body’s dominant side (uses a knife) with the side that handles the product. To perform the McNemar test (*n* = 100), the task “Chicken slaughter” was excluded, since it was the only one that had one worker and the risk level remained equal in both conditions, which made the test unfeasible.

The Pearson correlation was used to verify the relationship between the OCRA scores in the two sharpness conditions and the number of gloves, technical action frequency, posture, force and frequency factors, plus the scores of the Borg scale and each bodily joint. The cycle time and the number of technical actions per cycle were correlated as well. For all tests, a statistical significance level of *p* ≤ 0.05 was adopted and, the correlation classification of Hinkle et al. [34] was implemented.

## 3. Results

Among the 18 repetitive tasks in which workers used a knife to cut the chickens (Table 4), the waiting time (not doing technical actions—cutting) was more than 50% of the cycle time in only two tasks, but the cycles were identical to each other (Removing breast/removing condemnations and Chicken slaughter tasks). For this reason, the force factor values were lower for these tasks. Most workers conducted the tasks in cold environments (91%) and performed job rotation schemes (58%), of which 50% were formed by four tasks (in at least one task the worker used a knife).

Of the 101 workers, 50% mentioned that the work pace was routinely high. This was confirmed since 97% of them performed their tasks with high frequency (7–10 scores), and 99% submitted cycle times less than 30 s (high repeatability). Moreover, there was a high correlation between the cycle time and the number of technical actions per cycle (r = 949; *p* < 0.001). When analyzing the task stereotypies, the majority of the workers completed tasks that received a score of 3 (91%), as the cycle time was less than 8 s (40% of the tasks) or repeated identical movements were carried out almost the entire cycle time.

Considering the hand that used a knife, the workers accomplished 69.1 ± 13.3 technical actions per minute (high frequency), and the task that presented the greatest frequency was “deboning leg—bone 2” (88.3) and the task with the lowest frequency was “Chicken slaughter” (20.7) (Table 4). It should be noted that in 94% of the tasks, one common additional risk factor was present (work completely determined by machines).

Through the Borg scale, the range of workers’ perceived effort with a sharp knife was 0.5 to 4 and between 2 and 8 with a dull knife. For the “well-sharpened” condition, most of the workers assigned the cutting force as extremely light, very light and light (scores 0.5–2) (87%), though 37% of the analyses received a score of 0 in the force factor (Borg scale versus fraction of the time). On the other hand, if the same tasks were performed in the “poorly sharpened” condition, the scores changed to hard and very hard (scores 5–8) (65%), which caused the saturation of the force factor score (24 or 32 points) in 64% of the workers (Table 5).

The OCRA Checklist median score showed that cutting with a poorly sharpened knife was greater (50.60) than with a well-sharpened knife (24.04) (*p* < 0.001). In both knife conditions of “sharp” (70%) and “dull” (91%), most tasks presented a high risk of developing UL-WMSD in the workers (OCRA score > 21.5%). Consequently, this result caused a significant increase in the risk level of UL-WMSD, since 29% workers who were submitted to a moderate risk changed to a high risk when the knife was dull (*p* < 0.001). Only one task remained at low risk in both conditions (Chicken slaughter) (Table 6).

By comparing the dominant upper limb (right) in the “sharp” knife condition with the contralateral side, it was verified that the OCRA median score of the right side (knife) (24.04) was significantly lower than the left side (products) (25.30) (*p* < 0.001). The OCRA score of the left side was 25.02 ± 2.67 (high risk) and most tasks (91%) received scores of 8, 9 or 10 for the frequency factor (67.88 ± 10.29 technical actions/min). Additionally, several tasks obtained a score of 2 for the force factor (90%), 1 for shoulder posture (87%), 0 for elbow (54%), 2 for wrist (74%) and 4 for hands-fingers (100%). This body side received a score of 3 for the stereotypy in 74% of the tasks and 2 for the additional factor in 97%.

Nevertheless, the highest scores attributed to the posture in the right side were 4 for the wrist (88%) and 3 for knife hand (67%). Considering that of all the scores calculated for the joints, only one was used to determine the posture factor (highest). It was shown that 57% of workers obtained the highest score in the wrist (score 4) and the hand with 11% (score 3) and 1% (score 4), however, 32% of them received the same score (score 4) for both joints.

Most workers mentioned feeling bodily discomfort in the upper limbs (64%), mainly in the shoulder (41%), wrist (12%), arm (5%), forearm (4%), fingers (3%), elbow (2%) and hand (1%). Of those workers who reported discomfort caused by knife use (27%), most felt it in the shoulders (74%), wrist (37%), but only a few felt it in the hands (4%), forearm (4%) and fingers (8%). The cold was felt in at least one hand by 39% of workers, despite 58% having noticed the cold hands. To minimize exposure to cold, workers protected the hand using the knife with one (46%) or two (45%) gloves. There was no correlation between the OCRA scores in the two sharpness conditions and the number of gloves in the dominant hand, this may have occurred because workers wore only one or two gloves.

Finally, there was a high correlation between the OCRA Checklist in the “well-sharpened” condition and the frequency factor (r = 0.706; *p* < 0.001); moderate correlation with the Borg scale (r = 0.568; *p* < 0.001), posture factor (r = 0.589; *p* < 0.001), force factor (r = 0.632; *p* < 0.001) and frequency of technical actions (r = 0.688; *p* < 0.001). Nonetheless, with wrist (r = 0.248; *p* = 0.013) and hand-finger postures (r = 0.287; *p* < 0.001), there were weak correlations. Most results showed that 40% of the score variation of the OCRA in this condition was explained by the force, 50% by the frequency and 35% by the posture factors. Whereas in “poorly sharpened” conditions, a 97% score variation of the OCRA was explained by the force factor (r = 0.983; *p* < 0.001; high correlation), however only 7% by the frequency factor (r = 0.269; *p* = 0.007; weak correlation).

## 4. Discussion

The OCRA Checklist score had significant correlations with the frequency factor in both sharpness conditions, but it was higher with the well-sharpened knife. The current work corroborates previous research, since the average of repetitive actions performed by poultry slaughterhouse workers was high (59.1, 63.7, 64.4, 79.8, 75.5 actions/min, respectively); and represented 8 to 10 points on the OCRA scale (0–10) [35,36,37,38,39]. Nonetheless, although the results are similar to the present study (69.1 actions/min = 10 points), these studies did not determine the number of technical actions, specifically for groups of cutting tasks. Depending on the cutting task demand, it is notable that the risk exposure of the worker may be greater. According to Colombini and Occhipinti [5], if the knife tip is positioned before cutting, this technical action must also be counted, increasing the number of technical actions per minute.

In addition to the worker being exposed to a high work pace in slaughterhouses, they use tools since most of the exported products are cut. The use of knives in food factories has led many authors to make recommendations, citing that sharp knives could be provided near the line so employees can change out when they feel a sharper knife is needed [6]. The worker who executes cutting tasks in a slaughterhouse, usually has a steel polisher or a knife sharpener or more rarely, a worker polishes the edge of the knives at the workstation throughout the workday. The Brazilian regulatory norm NR-36 mentions that employers must train the workers in knife sharpening. Additionally, the organization of the process and the speed of the production line must consider the temporal variability required by different production and product demands, thus, the time necessary to attend to knife sharpening/polishing must be calculated as well [40]. It is noteworthy that in this research, the technical actions of knife polishing by the workers, were disregarded, but even so, the technical action frequencies were high.

Most cycle times were 8 s or less and had more than 30 technical actions per min. Kilbom [41] concluded that a work is repetitive if the duration of the work cycle is below 30 s and rates of 25–33 movements per minute should not be exceeded in order to prevent tendon disorders. This author also cites that if the force requirements are high, these rates must be lowered. Likewise, after analyzing 32 job tasks of a poultry processing plant, a study proved that 81% of the jobs were greater than the American Conference of Governmental Industrial Hygienists—Action limit, and 59% were above the Threshold limit value for hand activity and force [6].

Unlike other studies, in which the risk of developing UL-WMSD was moderate (predicts 10.6 to 21.5% of the workers) [35,36,38,39,42,43], this paper found high risks in the two conditions of knife sharpness (>21.5%), corroborating with the research of Reis et al. [37,38].

The left side of the body presented a greater risk compared to the right, contrary to studies that also included tasks without the use of tools [37,39]. As reported by Colombini and Occhipinti [5], for standard meat cutting operations, the left side may be at higher risk than the right side, since the left hand is holding the meat (pinch or palmar grasp) while the right hand is holding the knife (grip).

One paper regarding job rotations states that the force applied by slaughterhouse workers was moderate in 71% of tasks and for 1/3 of the cycle time (scored 2) [39]. Conversely, an investigation of all production sectors in seven poultry slaughterhouses with 995 workers found that 56.1% achieved “heavy or moderate” force [44]. Furthermore, a survey with workers from the cutting sector in a poultry slaughterhouse perceived the applied exertion when cutting meat as light (score 2) (48.7%) and moderate (score 4) (42.1%) and 63.1% perceived their knives as very sharp [26]. These results differ from the present study, as with a “well-sharpened” knife, 50% of the workers identified the exertion as light (score 2) and 37% as very light and extremely light (score 0.5–1). Already with a “poorly sharpened” knife, the scale points were hard and very hard, and it saturated the force factor for most workers, which would significantly increase the risk of UL-WMSDs. According to Reis et al. [36,37,38], reducing the risk level of some tasks in slaughterhouses, by only decreasing the work rate, was not an efficient intervention due to the high demand for strength required to perform them.

Although there are inconclusive results on the effects of knife sharpness on cutting force [15,21], several papers indicate that the use and maintenance of sharp knives reduce the risk of WMSDs [4,20,27,45], as it requires less force to cut [6,22].

Therefore, slaughterhouses follow regular knife sharpening protocols (four times per work shift), as well as provide training in the use of knife sharpening accessories at the workstation, the OCRA method evaluator should not assume that the knives are well sharpened. Since the method recommends that the workers must be asked to assign their perceived exertion with the Borg scale on the actions that require the use of force [5].

The negative influences of a “poorly sharpened” knife were confirmed by the OCRA method, since there was a significant increase in the risk of UL-WMSDs due to the biomechanical overload needed to cut. Endorsing this paper, OSHA [3] cited that when using dull knives, the workers must apply more force than necessary to get the job done. Therefore, it is recommended that employees use only sharp knives, and the employer ensures that the knife change-out schedule is strictly followed [6,40]. Regardless, Szabo et al. [45] revealed that significant increases in force may be anticipated for infrequent reconditioning, which may escalate fatigue onset and the risk of WMSDs. Savescu et al. [28] cited that to improve MSD prevention, sharpening and steeling operations should not be considered as independent activities, but taken into account as a continuity of working actions.

One assessment established a high prevalence (34%) of poultry-processing workers with evidence of carpal tunnel syndrome, moreover, over half of the participants reported hand or wrist symptoms (58%) [6]. Contrarily, this and other evaluations have shown that several poultry slaughterhouse workers perceived discomfort in the shoulder [26,46,47,48].

Like this study (64% of workers), Tirloni et al. [26] verified that most poultry slaughterhouse workers felt discomfort in the upper limbs (54%). Nonetheless, analyzing discomfort globally, Pinetti et al. [46] showed that 43% of respondents had symptoms in at least one body region, however, Tirloni et al. [12,47] presented superior values (67.2% and 71.5%, respectively). A paper proved that there was a significant association (*p* < 0.05) between perception of bodily discomfort in slaughterhouse workers and performance of repetitive tasks (OR = 1.81) and perception of cold (OR = 2.05) [12].

In addition to using a knife, employees worked in a cold environment, manipulated refrigerated products and most of them wore overlapping gloves (1 to 2). A study demonstrated that poultry slaughterhouse workers (143) wore one to five gloves on each hand, and the majority wore three overlapping gloves (57.3%). As reported by Willms et al. [49], perceived exertion significantly increased as a function of glove thickness. Additionally, decreases in maximum grip force compared with bare hands were observed for the thickest glove. Gloves are indispensable for preventing accidents but make it harder to grip objects and may become slippery, requiring even more force [5]. Despite this, there was no correlation between the number of gloves used and the perceived exertion of the workers when cutting the product (*p* ≤ 0.05) [26].

Notwithstanding, the use of several overlapping gloves was insufficient in promoting thermal insulation of the hands, since most slaughterhouse workers presented at least one finger with temperatures of ≤15 °C (66.4%) and ≤24 °C (99.3%), perceived their cold hands, and wore three overlapping gloves (57.3%) [11]. However, a positive factor is that the hand that holds the knife is warmer than the hand that handles the product [10,11,50]. In this study, a smaller percentage felt cold (39%), but most noticed cold hands (58%). Tirloni et al. [11] assessed that there was an association between feeling cold in the hands and perceiving cold hands (*p* < 0.001), in which most workers who perceived their hands as cold felt cold in their hands (66.3%).

Dias et al. [51] analyzed cutting tasks and verified that most of them exhibited a score for the hand or wrist postures higher than the other joints, similar to the current paper. In another study, the highest scores for bodily posture on the OCRA also occurred in the hand-fingers with a significantly positive correlation to the OCRA scores of “with job rotation—tasks < 1 h” [39]. The grip of the hand holding the knife is not optimal, e.g., when the index finger is extended forward to guide the tip of a knife or screwdriver [5]. Additionally, the bad grip posture, as noted in this research, lasted almost the entire cycle time. According to Arvidsson et al. [52], cutting tasks require significant efforts of flexion and extension of the wrist, and as mentioned by OSHA [3], workers carry out exertions to maintain control of equipment or tools.

Overall, it is observed that three risk factors of the OCRA methods were high: the work rate (frequency factor), 3.5 h without periods of physiological recovery (recovery multiplier) and the work shift duration—8 h 48 min, consequently, the repetitive work time as well (duration multiplier). These factors may have interfered in the results of the “well-sharpened” condition. Therefore, an intervention in only one risk factor (force) is not enough to minimize the risk of UL-WMSD in these slaughterhouses. In addition, effectiveness of employer-dictated work practices and policies to reduce or prevent hazardous exposures depends on employer commitment and employee acceptance. For this, regular monitoring and reinforcement must be performed to ensure they are followed consistently [6].

### Strengths and Limitations

The paper’s limitations were that the workers were not interviewed about knife sharpness conditions at the time of data collection. The recall method was used to evaluate the perceived effort in different knife conditions, which may have caused memory bias. Conversely, as a strength, this was an exploratory study and the first one to analyze the influence of knife sharpness on the subjective perceptions of cutting force in poultry slaughterhouse workers applying the OCRA method.

## 5. Conclusions

It was concluded that in both sharpening conditions analyzed, most of the tasks presented a high risk in developing UL-WMSD. As the work rate was high, there were awkward postures of the wrist and hand/fingers (knife side), the machine set the pace, and not all work hours were recovered with rest breaks. Half of the workers perceived the work pace as routinely high; tasks had high repeatability, due to the raised frequency of technical actions and cycles less than 30 s. In the “well-sharpened” condition, most workers assigned the force to cut as light, very light and extremely light, unlike the “poorly sharpened” condition, which was hard, very hard, saturating the force factor. Hence, the OCRA scores revealed that the expected prevalence of UL-WMSDs in workers who cut with a dull knife was significantly greater than with a sharp one. Furthermore, numerous poultry slaughterhouse workers reported feeling bodily discomfort in the upper limbs, especially in the shoulders and wrists, and perceived their hands as cold.

The slaughterhouse workers who performed cutting tasks were vulnerable to ergonomic hazards by highly repetitive movements, inadequate postures of the wrist and hand, besides, the risk increased when the knife was not sharpened properly. Thus, ergonomists and occupational health and safety teams must include knife sharpening in the standard operating procedure in slaughterhouses. This measure aims to promote the maintenance of knife sharpening throughout the workday, avoiding the application of unnecessary force and the development of UL-WMSD.

## Figures and Tables

**Table 1:** Work organizational characteristics in slaughterhouses.

Description	Slaughterhouse 1	Slaughterhouse 2	Slaughterhouse 3	Total
Total workers (*n*)	2300	1130	3100	6530
Workers in productive area	1600	930	2500	5030
Participants (*n*)	19	36	46	101
Chickens slaughtered daily	300,000	115,000	280,000	

**Table 2:** Subjective assessment of perceived exertion perception by Borg scale.

0	0.5	1	2	3	4	5	6	7	8	9	10
Missing	Extremely light	Very light	Light	Moderate	Moderate +	Hard (heavy)	Hard +	Very hard	Very hard +	Very hard ++	Extremely hard (almost max.)

**Table 3:** Force factor score using the “fraction of the time” and the Borg scale.

Borg Scale	Fraction of the Time and Force Factor				
The working activity requires:	<1/3 of time	≈1/3 of time	≈1/2 of time	≈2/3 of time	≈all the time
MODERATE FORCE (score 3–4)	1	2	4	6	8
	peaks of 1–2 s every 10 min	≈1% of time	≈5% of time	≈10% of time or more	-
INTENSE FORCE (score 5–6-7)	4	8	16	24	-
Almost MAXIMAL FORCE (score 8 or more)	6	12	24	32	-

**Table 4:** Description of the cutting tasks for the body’s dominant side.

Slaughterhouses	Tasks (18)	Cycle Time (s)	Technical Actions/Cycle	Technical Actions/min	Frequency Factor	Time/One Action	*n* (%) Workers	OCRA Checklist—Sharp Knife	OCRA Checklist—Dull Knife	OCRA Score Increase (%)
1	Cleaning breast	15.3	16.7	65.5	9	0.92	10 (53)	25.81 ± 2.0	46.55 ± 13.29	80.4
	Deboning leg—bone 1	10.1	14.7	87.3	10	0.69	4 (21)	25.93 ± 2.28	41.75 ± 13.52	61.0
	Deboning leg—bone 2	8.7	12.8	88.3	10	0.68	5 (26)	27 ± 1.91	39.22 ± 13.52	44.9
2	Removing neck	2.6	2	46.2	5	1.30	1 (3)	22.77 ^†^	48.07 ^†^	111.1
	Removing back	4.6	3	39.1	4	1.53	1 (3)	24.04 ^†^	24.04 ^†^	0.0
	Deboning whole leg	16.4	23	84.1	10	0.71	16 (44)	26.88 ± 2.91	51.23 ± 10.63	90.6
	Cutting Kakugiri	14.8	21	85.1	10	0.70	2 (6)	20.87 ± 1.79	24.67 ± 0	18.2
	Cleaning breast	17.9	16	53.6	7	1.12	6 (17)	18.13 ± 3.72	42.59 ± 11.29	134.9
	Cleaning breast—automatic	6.1	7	68.9	10	0.87	6 (17)	25.72 ± 1.31	45.12 ± 17.94	75.4
	Removing wing	4.7	6	76.6	10	0.78	3 (8)	24.04 ± 3.31	36.69 ± 13.83	52.6
	Deboning sassami	5.5	4	43.6	5	1.38	1 (3)	20.24 ^†^	48.07 ^†^	137.5
3	Removing wing	2.1	3	85.7	10	0.70	6 (13)	25.3 ± 3.23	53.55 ± 17.62	111.7
	Deboning back	40	50	75	10	0.80	1 (2)	21.51 ^†^	49.34 ^†^	129.4
	Removing breast/removing condemnations ^#^	5	5	60	8	1.00	9 (20)	22.49 ± 0.84	30.08 ± 12.36	33.7
	Cleaning breast	5.4	5.6	62.2	8	0.96	11 (24)	21.16 ± 2.54	41.4 ± 14.94	95.7
	Cleaning leg	12.4	12.4	60	8	1.00	17 (37)	22.55 ± 2.38	45.91 ± 14.28	103.6
	Chicken slaughter ^#^	2.9	1	20.7	0	2.90	1 (2)	12.65 ^†^	13.92 ^†^	10.0
	Removing back	3.6	4	66.7	9	0.90	1 (2)	27.83 ^†^	22.66 ^†^	100.0
Range	3 to 8 tasks/slaughterhouses	2.1 to 40	1 to 50	20.7 to 88.3	0 to 10	0.68 to 2.90	101	12.65 to 29.10	13.92 to 64.52	0 to 137.5
Average ± SD	-	10.6 ± 5.9	12.4 ± 7.7	69.1 ± 13.3	8.7 ± 1.5	0.91 ± 0.25	-	23.79 ± 3.10	43.57 ± 13.51	83.7 ± 30.3

^#^ Less than 50% of cycle time by cutting; ^†^ OCRA Checklist value in condition of one worker performing the task; *n* (%) workers in relation to total workers per slaughterhouse: slaughterhouse 1 (*n* = 19), slaughterhouse 2 (*n* = 36), slaughterhouse 3 (*n* = 46); average/SD regarding the 101 workers.

**Table 5:** Perceived exertion of workers in relation to the knife sharpness conditions.

Borg Scale (Score)	Sharpness Conditions	
	Sharp (%)	Dull (%)
0.5	12	-
1	25	-
2	50	2
3	12	18
4	1	16
5	-	33
6	-	17
7	-	10
8	-	5
**Force Factor**		
0	37	-
1	6	1
2	45	8
4	12	27
24	-	59
32	-	5

*n* = 101 workers; Borg scale and Force factor by OCRA Checklist method [5].

**Table 6:** Risk assessment of repetitive upper limb movements in sharpness conditions.

Risk Level	OCRA Checklist Score	Incidence of UL-WMSDs (%)	Sharpness Conditions (*n*)	
			Sharp	Dull
Acceptable	<7.5	< 5.3	0	0
Borderline or very low	7.6–11	5.3–8.4	0	0
Low	11.1–14	8.4–10.8	1	1
Moderate	14.1–22.5	10.8–21.5	30	1
High	>22.5	>21.5	70	99

*n* = 101 workers; Risk classification and score by OCRA method [5].
